# Evaluating the rhizospheric and endophytic bacterial microbiome of pioneering pines in an aggregate mining ecosystem post-disturbance

**DOI:** 10.1007/s11104-022-05327-2

**Published:** 2022-03-09

**Authors:** Kiran Preet Padda, Akshit Puri, Nguyen Khoi Nguyen, Timothy J. Philpott, Chris P. Chanway

**Affiliations:** 1 Department of Forest and Conservation Sciences, Faculty of Forestry, The University of British Columbia, Vancouver, BC Canada; 2 Present Address: School of Agriculture and Food Science, University College Dublin, Belfield, Dublin 4, Ireland; 3 UCD Earth Institute, University College Dublin, Belfield, Dublin 4, Ireland; 4 Microbiome Insights Inc., Vancouver, Canada; 5 British Columbia Ministry of Forests, Lands and Natural Resource Operations, Williams Lake, BC Canada

**Keywords:** Acetic acid bacteria, Endophytes, Nitrogen, Pinus, Rhizobiales, Tree microbiome

## Abstract

**Aims:**

Despite little soil development and organic matter accumulation, lodgepole pine (*Pinus contorta var. latifolia*) consistently shows vigorous growth on bare gravel substrate of aggregate mining pits in parts of Canadian sub-boreal forests. This study aimed to investigate the bacterial microbiome of lodgepole pine trees growing at an unreclaimed gravel pit in central British Columbia and suggest their potential role in tree growth and survival following mining activity.

**Methods:**

We characterized the diversity, taxonomic composition, and relative abundance of bacterial communities in rhizosphere and endosphere niches of pine trees regenerating at the gravel pit along with comparing them with a nearby undisturbed forested site using 16S rRNA high-throughput sequencing. Additionally, the soil and plant nutrient contents at both sites were also analyzed.

**Results:**

Although soil N-content at the gravel pit was drastically lower than the forest site, pine tissue N-levels at both sites were identical. Beta-diversity was affected by site and niche-type, signifying that the diversity of bacterial communities harboured by pine trees was different between both sites and among various plant-niches. Bacterial alpha-diversity was comparable at both sites but differed significantly between belowground and aboveground plant-niches. In terms of composition, pine trees predominantly associated with taxa that appear plant-beneficial including phylotypes of *Rhizobiaceae*, *Acetobacteraceae*, and *Beijerinckiaceae* at the gravel pit and *Xanthobacteraceae*, *Acetobacteraceae*, *Beijerinckiaceae* and *Acidobacteriaceae* at the forest site.

**Conclusions:**

Our results suggest that, following mining activity, regenerating pine trees recruit bacterial communities that could be plant-beneficial and support pine growth in an otherwise severely N-limited disturbed environment.

**Supplementary Information:**

The online version contains supplementary material available at 10.1007/s11104-022-05327-2.

## Introduction

Forest landscapes around the world are subject to alteration by natural and anthropogenic disturbances, often leading to ecosystem degradation (Caviedes and Ibarra 2017). Soil health is one of the key attributes of forest ecosystem affected by degradation. Disturbance regimes like resource extraction degrade forest soils through compaction and displacement of litter and soil, which not only affect the availability of nutrients in soils and inhibit root growth but also obstruct the supply of oxygen and water to soil microbes and plant roots (Osman 2013). These impacts can thus alter the plant–soil–microbial dynamics and subsequently affect the normal ecosystem functioning (Bowd et al. 2019). In Canada, mining has been identified as one of the major factors disrupting forest ecosystems (LeMay 1999; Frelich 2014). One of the top non-fuel mineral commodities mined in Canada includes mineral aggregates – crushed stone, rocks, gravel, and sand (Koehnken 2018; Barry 2019). The establishment of pits and quarries for aggregate mining requires complete removal of natural vegetation and topsoil or in some cases even subsoil (Winfield and Taylor 2005), resulting in the loss of the existing biodiversity as plant and soil habitats are destroyed. Artificial reclamation of such disturbed systems is one way to remediate ecosystem services, but it may involve costly procedures and the risk of introducing exotic floral and faunal species (Macdonald et al. 2015). However, when such sites are left unreclaimed, the nearby biological communities may take precedence and play a crucial role in the natural restoration of ecosystem health and productivity. Yet, our understanding is largely limited regarding how the assemblages of these pioneering aboveground and belowground biological communities have evolved to thrive in these disturbed ecosystems.

Lodgepole pine (*Pinus contorta var. latifolia*) is an important gymnosperm tree species native to western North America that has the remarkable ability to withstand severe natural and anthropogenic disturbances and thrive on fire-affected sites, dry and coarse sandy soils, road cuts, and mining pits (Lotan and Critchfield 1996; Chapman and Paul 2012; Puri et al. 2018; Turner et al. 2019). Culture-based studies suggest that lodgepole pine trees associate with endophytic plant-growth-promoting bacteria having various abilities including nitrogen (N) fixation, phosphate solubilization and stress tolerance to grow on dry, coarse, nutrient-poor soils (Puri et al. 2020a, b). Swift recovery of soil N pools, significant growth of lodgepole pine trees and optimal tissue N contents have been observed in a lodgepole pine dominated stand severely affected by fires in Yellowstone National Park, USA (Turner et al. 2019). The absence of known nodulating plants and minimal deposition of atmospheric N in these stands indicate the unique ability of lodgepole pine trees to thrive under extreme conditions, possibly by forming mutualistic associations with their microbial partners to fix N (Turner et al. 2019; Chapman and Paul 2012).

The soil and plant microbiome has been a growing area of research with several studies establishing the key role of microbial communities in plant growth and survival (Compant et al. 2019). Although the rhizospheric bacterial communities and their interactions with plants have been studied widely for the last few decades, the endophytic bacterial microbiome has only received recent attention with studies suggesting that they could be better protected from external abiotic and biotic stresses due to their niche inside the plant tissues (Chanway et al. 2014). Most of our understanding about the overall plant bacterial microbiome (endophytic + rhizospheric) has been derived from model plants such as *Arabidopsis* or agricultural plants (Rosenblueth and Martínez-Romero 2006; Reinhold-Hurek and Hurek 2011), whereas the tree microbiome has been an underexplored field of research (Pirttilä and Frank 2018). Considering the long life-span and large biomass of conifers, bacterial communities may play an even more important role in helping these trees to thrive in the extreme environmental conditions that characterize boreal and temperate forest ecosystems, including the weakly-developed nutrient-poor soils, slow mineralization rates, climate extremes, and invasive pests (Puri et al. 2017a; Pirttilä and Frank 2018). For instance, bacterial communities in the foliage of North American conifers such as coast redwood (*Sequoia sempervirens*), giant sequoia (*Sequoiadendron giganteum*), and limber pine (*Pinus flexilis*) have been reported to largely comprise taxa closely related to N-fixing acetic acid bacteria (Carrell and Frank 2015; Carrell et al. 2016; Moyes et al. 2016). It was suggested that associating with these foliar endophytic communities may be an evolutionarily stable N-fixing strategy of conifers to thrive on disturbed, N-poor soils, and may explain how boreal and temperate forests accumulate more N than can be accounted for by known N input pathways (Wurzburger 2016).

Of the various conifer species, the highly adaptable nature of lodgepole pine may be attributed, at least in part, to its microbial partners as evidenced previously in culture-based inoculation studies (Anand et al. 2013; Padda et al. 2019). Chapman and Paul (2012) provided compelling evidence using the ^15^N natural abundance technique that lodgepole pine trees growing on abandoned gravel mining pits in the central interior of British Columbia (BC), Canada could be accessing biologically fixed N in conjugation with certain symbionts. In addition, lodgepole pine trees at the gravel pits had virtually identical growth rates in terms of tree height, leader length and root collar diameter when compared with the lodgepole pine trees of equivalent age from a nearby undisturbed forest stand having intact soil (Chapman and Paul 2012). Similar tree growth rates despite drastic differences in edaphic conditions raise the possibility that pioneering lodgepole pine trees could rely on their microbiome for survival and fitness under difficult environmental conditions.

In this study, our primary objective was to examine the bacterial communities inhabiting the rhizosphere and endosphere of lodgepole pine trees growing at an aggregate mining site (Skulow gravel pit) located in central-interior BC and suggest their potential role in tree growth and survival following mining activity. Additionally, we determined if these pioneering pines assemble similar or different bacterial microbiomes post-disturbance by comparing them with lodgepole pine trees from a nearby undisturbed forest stand along with assessing how bacterial communities differentiate among various tree niches (needle, stem, root, rhizosphere). Our secondary objective was to compare the nutrient content of soil and plant (lodgepole pine) samples collected from the gravel pit with the undisturbed forest site and to determine if various soil parameters affected the bacterial community structure of pine trees at both sites.

## Materials and methods

### Sampling sites

The Skulow pit is located 40 km north-east of Williams Lake (52°18′54.1” N, 121°53′39.3” W, 1064 m a.s.l.) in the central interior of BC. The average yearly temperature for 1981–2010 recorded at the nearby Camille Lake weather station was 3.7 °C and the normal annual precipitation was 174.1 cm as snowfall and 361.9 mm as rainfall (Government of Canada n.d.). Lodgepole pine trees were predominant inside and on the slopes of the Skulow pit, whereas hybrid white spruce (*Picea glauca* × *engelmannii*) and Douglas-fir (*Pseudotsuga menziesii*) trees were present on the edges. Lodgepole pine trees were thriving on the bare gravel substrate characterized by no topsoil or organic forest floor, weak profile development and no soil horizons. Ten young lodgepole pine trees (<5 years old and < 30 cm height) were randomly selected from different sections within the pit. Similarly, ten young lodgepole pine trees were randomly selected from the nearby undisturbed forest stand (within a 250 m radius around the pit) which consisted of lodgepole pine, hybrid white spruce and Douglas-fir trees. The soil in this forest stand consisted of intact forest floor and distinct soil horizons with no evidence of anthropogenic soil disturbance.

### Plant and soil sampling

The selected lodgepole pine trees from the gravel pit and nearby forest stand were carefully extracted ensuring that the root system was preserved. The intact root system of each tree was excavated carefully using a shovel to undercut the roots, followed by gentle hand digging to allow the gravel and sand to fall free of the roots. Gloves were worn at all times during the sampling. Sterility was maintained by cleaning the shovel and hands with 75% ethanol before and after sampling each tree to avoid any cross-contamination. The bulk soil loosely attached to the pine roots was removed through vigorous shaking. Each tree was placed in a sterile plastic bag and immediately transported to the laboratory on dry ice and processed within two days of sampling. Mineral soil samples were collected around each tree (within 30–40 cm radius) in four cardinal directions from 0 to 20 cm depth using an Oakfield probe at both the gravel pit and the nearby undisturbed forest stand. Samples collected in four cardinal directions were pooled to obtain one mineral soil sample around each tree.

### Soil and plant analyses

Mineral soil samples were air-dried and sieved through a 2 mm sieve to remove coarse fragments. Each mineral soil sample was further divided into two subsamples and analyzed to determine the physicochemical properties and nutrient status of the gravel pit and the undisturbed forest stand soils. Total C, N, S; available N (NH_4_^+^ and NO_3_^−^); mineralizable N; available P; pH in H_2_O and CaCl_2_; cation exchange capacity (CEC); organic matter; percent sand, silt and clay; and extractable macro- and micro-nutrients (Al, B, Ca, Cu, Fe, K, Mg, Mn, Na, P, S and Zn) were determined at the Analytical Chemistry Services Laboratory, BC Ministry of Environment and Climate Change Strategy, Victoria, BC, Canada. Five trees collected from each site were used to analyze plant nutrient contents. The roots of each tree were washed under running water to remove soil particles. Each tree was oven-dried at 70 °C and sent to the Analytical Chemistry Services Laboratory to determine total C, N, S and standard nutrients including Al, B, Ca, Cu, Fe, Mg, Mn, Mo, P, K, S and Zn. To compare the soil and plant nutrient concentrations between the undisturbed forest site and gravel pit, an analysis of variance (ANOVA) was performed using the statistical package SAS University Edition (SAS Institute Inc., Cary, NC, USA).

### DNA extraction and 16S rRNA sequencing

Tree bacterial microbiome analysis was performed using a metabarcoding approach to elucidate and compare the structure of bacterial communities present in the rhizosphere and internal tissues of young lodgepole pine trees growing in the gravel pit and the nearby undisturbed forest stand. Five pine trees from each site were used for the microbiome analysis. Once trees from each site were transported to the lab, rhizosphere soil samples (~ 1 g) were obtained from each tree by carefully collecting the soil particles intimately attached to the roots using a scalpel. Subsequently, each tree was surface-sterilized by immersing in 2.5% (w/v) sodium hypochlorite for 2 min, followed by three 30-s rinses in sterile distilled water (Puri et al. 2018). Needle, stem and root tissue samples (~250 mg) from each surface-sterilized tree were collected for subsequent DNA isolation.

Total genomic DNA from each needle, stem, root and rhizosphere soil sample was extracted using the Qiagen MagAttract PowerSoil DNA KF Kit following the manufacturer’s protocol. All DNA extractions were performed in triplicate per sample, after which extracts were combined into one sample. The polymerase chain reaction (PCR) was performed in triplicate to reduce PCR bias. Following the protocol outlined by Kozich et al. (2013), PCR amplification of the prokaryotic 16S rRNA genes was performed using dual-barcoded primers targeting the V4 region (515F 5’-GTGCCAGCMGCCGCGGTAA-3′, and 806R 5’-GGACTACHVGGGTWTCTAAT-3′). PCR conditions used for 16S sequencing were identical to those of Kozich et al. (2013) and Gweon et al. (2015), respectively. Amplicons were sequenced with an Illumina MiSeq using the 300-bp paired-end kit (v.3). Bacterial sequences were denoised, taxonomically classified using Silva (v. 138) as the reference database, and clustered into 97%-similarity operational taxonomic units (OTUs) with the mothur software package (v. 1.44.1) (Schloss et al. 2009), following the recommended procedure. Paired-end reads were merged and curated to reduce sequencing error (Huse et al. 2010). The potential for contamination was addressed by co-sequencing the DNA amplified from specimens with template-free control (negative control) and cloned Thioglobaceae SUP05 DNA (positive control). For both positive and negative controls, the extraction kit reagents were processed the same way as the specimens. The OTUs were considered putative contaminants (and were removed) if their mean abundance in controls reached or exceeded 25% of their mean abundance in specimens. The raw sequence data have been deposited in the NCBI Sequence Read Archive (BioSample accession no. SAMN19608371 and BioProject accession no. PRJNA736087).

### Bioinformatics and statistical analysis

Statistical analyses of bacterial community data were completed in R (version 3.6.2). Alpha diversity was estimated using the Shannon index on raw count OTU data after filtering out putative contaminants, compared across different groups by two-way ANOVA, and pair-wise comparisons were computed by Tukey post-hoc test. To estimate beta diversity across samples, we excluded OTUs occurring with a count of less than 3 in at least 10% of the samples and then computed Bray-Curtis indices. We visualized beta diversity, emphasizing differences across samples, using Principal Coordinate Analysis (PCoA) ordination. Differences in the communities of undisturbed forest and gravel sites were compared by permutational multivariate analyses of variance (PERMANOVA) with the site as the main fixed factor and using 9999 permutations for significance testing (Nguyen et al. 2020).

To identify differentially abundant taxa between the gravel pit and forest site, the DESeq2 package was used (Love et al. 2014). DESeq2 analysis was performed by taking the site (gravel pit vs undisturbed forest site) as a factor after normalizing the OTU data. To present the variation of bacterial microbiome profiles between the two sites and among different niches, the relative abundance (RA) of the 50 most abundant taxa at OTU level were visualized using ComplexHeatmap r package (Gu et al. 2016). Both rows and columns were unsupervised clustered based on distance matrix converted from Spearman’s correlation matrix (Nguyen et al. 2020) using the Ward.D method (Ward 1963), resulting in clusters of co-occurring bacteria. A dendrogram for columns was rendered to group individual samples based on their bacterial microbiome profile (dendextend r package v1.14.0; Galili 2015). Metadata such as site and niche were annotated and all analyses were performed in the R environment. The association between various soil properties and the bacterial community data was evaluated using phylogenetic isometric log-ratio (Silverman et al. 2017) and visualized using principal component analysis (See Supplementary file for detailed methodology).

## Results

### Soil and plant analyses

**Fig. 1:**
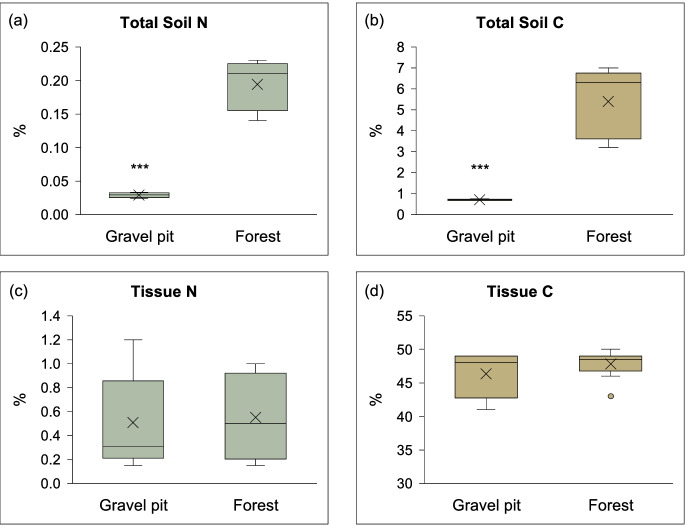
Boxplots of (**a**) total N and (**b**) total C (%) in the mineral soil samples (0–20 cm depth; n = 20) collected from the gravel pit and forest site. Boxplots of (**c**) tissue N and (**d**) tissue C (%) in the lodgepole pine tree samples collected from the gravel pit and forest site (n = 5). ‘╳’ represents the mean value on boxplots. *** P < 0.001 (significantly different from forest site)

**Fig. 2:**
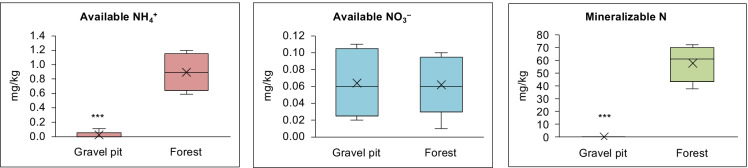
Boxplots of (**a**) available ammonium (**b**) available nitrate, and (**c**) mineralizable nitrogen present in the mineral soil samples (0–20 cm depth; n = 20) collected from the gravel pit and forest site. ‘╳’ represents the mean value on boxplots. *** P < 0.001 (significantly different from forest site)

Total N and C (%) in the mineral soil samples at the gravel pit were significantly lower (~7-fold) than the undisturbed forest stand (Fig. 1a and b). The amount of available NH_4_^+^ was >40-fold lower at the gravel pit in comparison to the undisturbed forest site, but no difference was observed in the available NO_3_^−^ content (Fig. 2a and b). Mineralizable N was also significantly lower (230-fold) at the gravel pit than at the undisturbed forest site (Fig. 2c). No available P was detected in the soil samples from the gravel pit whereas undisturbed forest soils had a considerable amount of available P (Table S1). Soil organic matter content was also significantly lower (9-fold) at the gravel pit (Table S1). Likewise, concentrations of several macro- and micro-nutrients were also significantly lower at the gravel pit compared to the undisturbed forest site (Table S1). Soil pH was acidic (4.9 to 5.5) at the forest site whereas gravel pit soils had a slightly alkaline pH (7.7 to 8.3) (Table S1). The sand content at the gravel pit (80%) was significantly higher than the forest soils (58%) (Table S1). Despite the large differences in the soil N contents at both sites, the tissue N contents (%) of lodgepole pine trees at the gravel pit and undisturbed forest site were identical (Fig. 1a and c). Similarly, no significant differences were observed in the tissue C content and macro- and micro-nutrient levels between pine trees originating from both sites (Fig. 1d; Table S2). The associations between various soil properties and the bacterial community were analyzed with phylogenetic isometric log-ratios and visualized using principal component analysis. At the undisturbed forest site, only available soil nitrate had a significant association (positive or negative) with the bacterial OTUs present in different niches of lodgepole pine trees (r^2^ = 0.383; p = 0.035) (Fig. S1). For the gravel pit, we determined that the soil properties had no significant association with the bacterial OTUs (Fig. S1).

### Bacterial community structure

**Fig. 3﻿:**
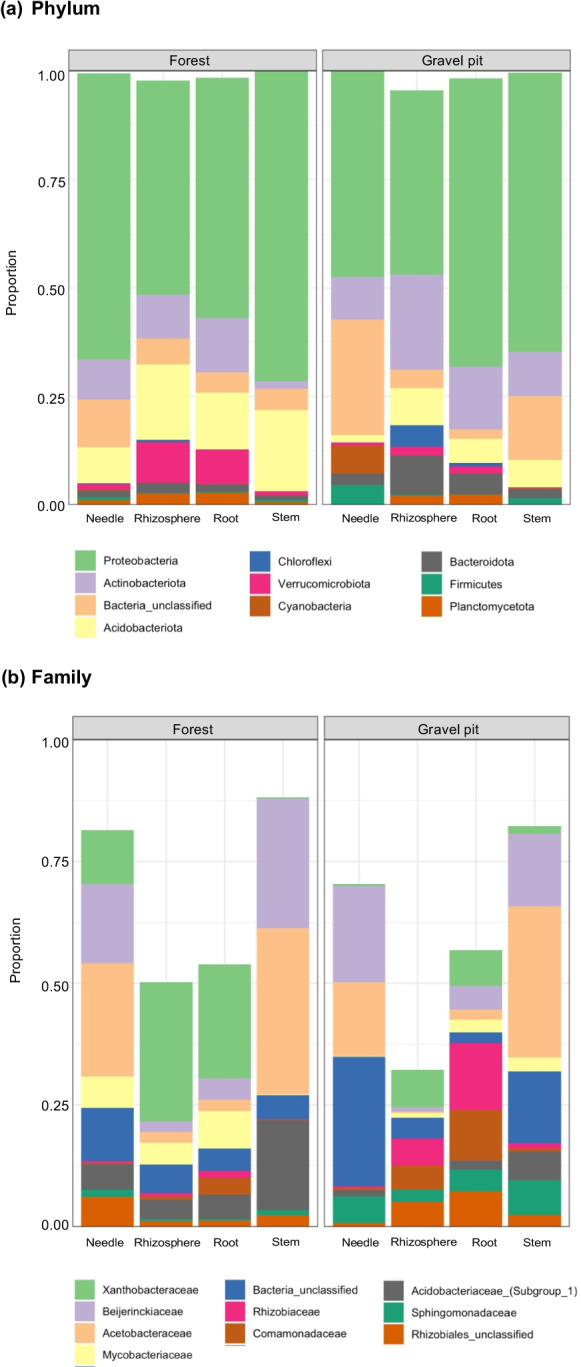
Relative abundance of bacterial (**a**) phyla and (**b**) families within individual lodgepole pine niches (needle, rhizosphere, root and stem) at the gravel pit and forest site. Low-abundance phyla (< 2% relative abundance) and low-abundance families (< 5% relative abundance) represent the unfilled portion of the bar plots

Considering taxa with a RA of ≥4%, members of the phylum *Proteobacteria* (65%) were the most abundant at the gravel pit, followed by the *Actinobacteriota* (13%), *Acidobacteriota* (7%), *Bacteroidota* (4%) and unclassified bacteria (4%) (Fig. S2). Similar to the gravel pit, *Proteobacteria* (60%) were the most abundant at the undisturbed forest site, but the proportion of *Acidobacteriota* (17%) was considerably higher in comparison to the gravel pit, followed by *Actinobacteriota* (8%), *Verrucomicrobiota* (5%) and unclassified bacteria (5%) (Fig. S2). Niche-wise comparison (rhizosphere, root, stem and needle) revealed that the proportion of *Proteobacteria* was higher in the aboveground lodgepole pine tissues (66–71%) as compared to the belowground rhizosphere and root tissues (49–55%) at the undisturbed forest site (Fig. 3a). In contrast, *Proteobacteria* were more abundant in the stem (64%) and root (66%) tissues of pine trees at the gravel site (Fig. 3a). *Actinobacteriota* were more abundant in the lodgepole pine rhizosphere (22%) at the gravel pit compared to the undisturbed forest (10%) whereas the proportion of *Acidobacteriota* was considerably higher in all niches at the undisturbed forest site in contrast to the gravel pit (Fig. 3a). *Alphaproteobacteria* were the most abundant class in all niches at both sites, dominated by the orders *Rhizobiales* and *Acetobacterales* (Fig. 3a and b). The *Acetobacteraceae* (acetic acid bacteria) was the most common bacterial family at the gravel pit, followed by the *Beijerinckiaceae* and *Rhizobiaceae* (Figs. 3b and S2) while at the undisturbed forest site, the *Acetobacteraceae*, *Xanthobacteraceae*, *Beijerinckiaceae* and *Acidobacteriaceae* dominated the bacterial taxa (Figs. 3b and S2).

#### Niche-wise comparison of Proteobacterial families

**Fig. 4:**
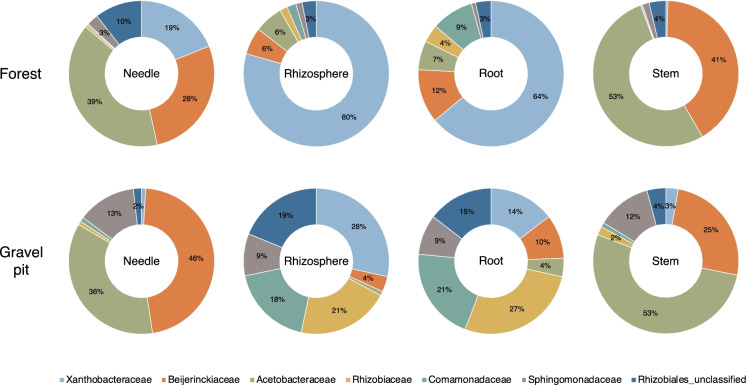
Relative abundance of families of Proteobacteria within individual lodgepole pine niches (needle, rhizosphere, root and stem) at the gravel pit and forest site. Only families with >4% relative abundance in at least one of the tissues are displayed on the doughnut charts

Given that the *Proteobacteria* was the most abundant phylum at both sites, we analyzed how different Proteobacterial families were distributed among different tree niches. Members of the *Xanthobacteraceae* heavily dominated pine roots and rhizosphere at the undisturbed forest site, whereas Proteobacterial families at the gravel pit were more evenly distributed, led by *Rhizobiaceae, Xanthobacteraceae and Comamonadaceae* (Fig. 4). Despite the substantial difference in belowground community composition at both sites, the aboveground needle and stem niches predominantly comprised of *Acetobacteraceae* and *Beijerinckiaceae* at the gravel pit and forest site (Fig. 4). However, the *Sphingomonadaceae* were more prevalent in pine needle and stem tissues at the gravel pit in comparison to the forest site (Fig. 4).

#### OTU distribution across different plant niches

**Fig. 5:**
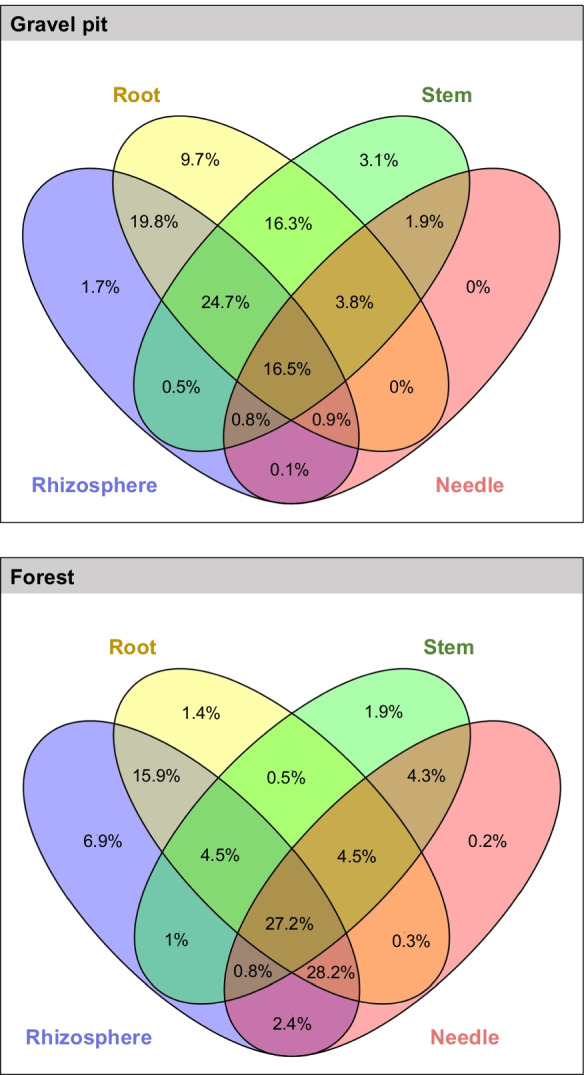
Venn diagram showing the distribution of OTUs across different lodgepole pine niches (needle, rhizosphere, root and stem) at the gravel pit and forest site. The diagram was created using VENNY 2.1 (https://bioinfogp.cnb.csic.es/tools/venny/index.html)

To provide a complete overview of the OTU distribution within the plants, we calculated the proportion of OTUs shared by the different plant niches (Fig. 5). The proportion of OTUs shared by all plant niches was 16.5% at the gravel pit and 27.2% at the undisturbed forest site. Of the total OTUs, 5% were exclusively observed in the aboveground endosphere niches (needle and stem) at the gravel pit and 6% at the undisturbed forest site whereas 25–30% of the total OTUs were exclusively found in the belowground rhizosphere and root niches at both sites. Additionally, we observed a higher overlap in OTUs between the rhizosphere and root samples (gravel pit: 19.8%, undisturbed forest: 15.9%) compared to the overlap between any other two niches.

### Alpha and beta diversity

**Fig. 6:**
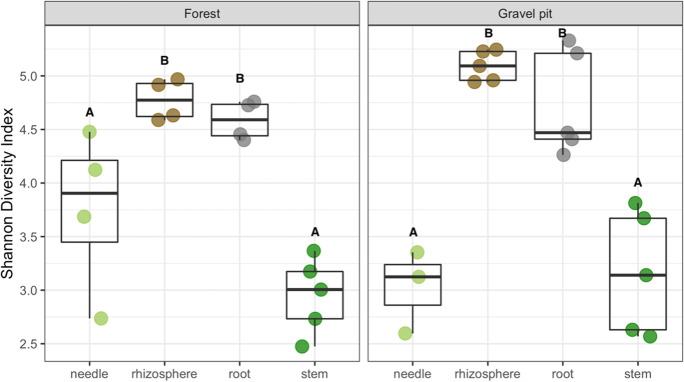
Shannon index showing alpha diversity of bacterial communities in different lodgepole pine niches (needle, rhizosphere, root and stem) at the gravel pit and forest site. Significant differences in Shannon index among various pine niches are indicated by different letters (P < 0.0001)

Alpha diversity based on the Shannon diversity index was comparable at both sites, with no significant difference between the undisturbed forest site and the gravel pit (Table 1). However, niche-wise, the rhizospheric and root bacterial communities of pine trees displayed a significantly higher Shannon diversity index than the stem and needle communities at both sites (P < 0.0001) (Fig. 6). Within the plant endosphere, the Shannon index was significantly higher in the root at both sites (P < 0.0001), whereas the stem bacterial community at the forest site had the lowest average Shannon diversity index (Fig. 6).

**Table 1:** Results of ANOVA for the effects of site (gravel pit and forest), niche (needle, stem, root and rhizosphere) and their interaction on Shannon diversity index

Treatments	df	F value	P value
Site	1	1.179	0.287
Niche	3	40.615	**0.000**
Site: Niche	3	2.284	0.102

Significant (P < 0.001) differences are in bold

**Fig. 7:**
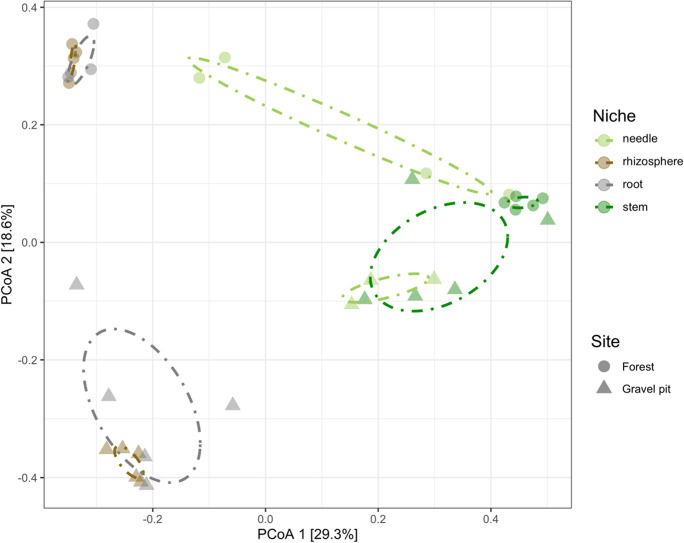
Bray-Curtis beta diversity of bacterial communities in different lodgepole pine niches (needle, rhizosphere, root and stem) at the gravel pit and forest site plotted using Principal-coordinate analysis (PCoA). Dash-dot lines represent the clustering of points for each niche of each site on the PCoA plot

Bray–Curtis beta diversity metrics with PCoA were used to visualize how the site (gravel pit vs undisturbed forest) and plant-niche impacted bacterial community composition (Fig. 7). The PCoA showed significant variability among different plant niches (P < 0.0001) as they formed distinct clusters away from each other along the axis (Fig. 7, Table 2). The site also had a significant effect on the clustering pattern (P < 0.0001), most noticeably in the roots and rhizosphere dataset (Fig. 7, Table 2). In addition, there was a significant site x niche interaction, where bacterial communities responded to the site as well as niche-type (Table 2).

**Table 2:** Effect of site (gravel pit and forest), niche (needle, stem, root and rhizosphere) and their interaction on bacterial beta-diversity assessed by PERMANOVA

Treatments	df	R^2^	P value
Site	1	0.1421	**0.0001**
Niche	3	0.3229	**0.0001**
Site: Niche	3	0.1290	**0.0001**

Significant (P < 0.001) effects are in bold

### Most abundant taxa

**Fig. 8:**
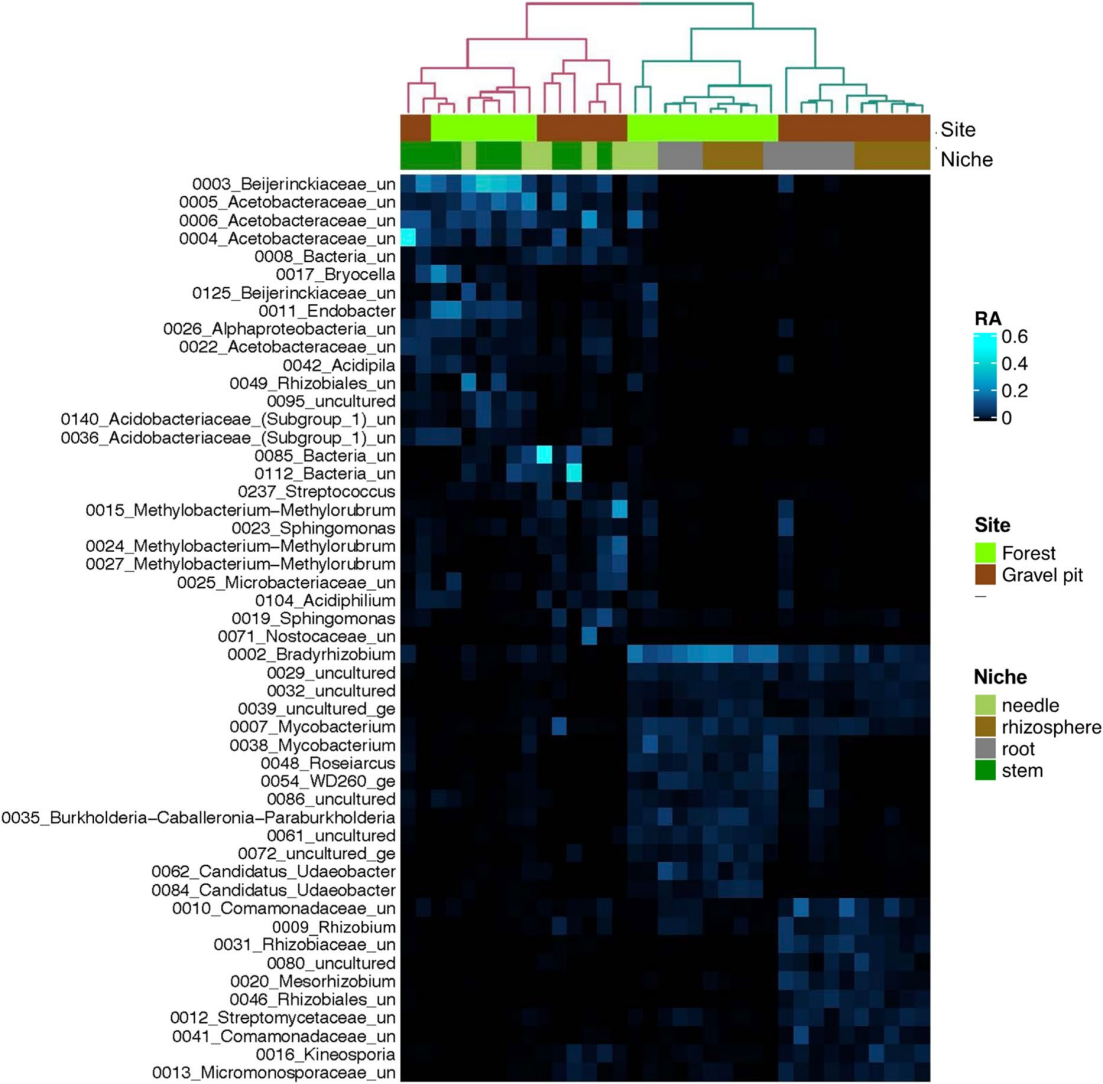
Heatmap showing the 50 most abundant OTUs in the entire bacterial community data set of gravel pit and forest site. Colour tones ranging from dark to light blue indicate lowest to highest relative abundance values. Rows and columns were unsupervised clustered based on the distance matrix converted from Spearman’s correlation matrix. A dendrogram for columns was rendered to group individual samples based on their bacterial microbiome profile

The RA of the 50 most abundant OTUs across all the samples showed that the most abundant OTUs, in general, tended to be dominant in either aboveground or belowground niches but not both (Fig. 8). Among the most abundant taxa, OTUs belonging to *Beijerinckiaceae* (6), *Acetobacteraceae* (5) and *Rhizobiaceae* (3) were predominant. OTU_3 of the *Beijerinckiaceae* family dominated multiple stem samples at the forest site with an RA of 30–40%. While OTU_2 (*Bradyrhizobium*) was prevalent in the forest root and rhizosphere samples (20% RA). Three OTUs of the *Acetobacteraceae* family dominated the needle and stem samples from both sites. In particular, the RA of OTU_4 (48%) was highest in the pine stem tissues from the gravel pit. Three OTUs of the genus *Methylobacterium* (*Beijerinckiaceae* family) dominated needle tissues at the gravel pit with an RA of up to 25%. Conversely, the belowground niches at the gravel pit were dominated by the *Rhizobiaceae* group, including *Rhizobium* and *Mesorhizobium* (up to 15% RA).

### Differentially abundant taxa

**Fig. 9:**
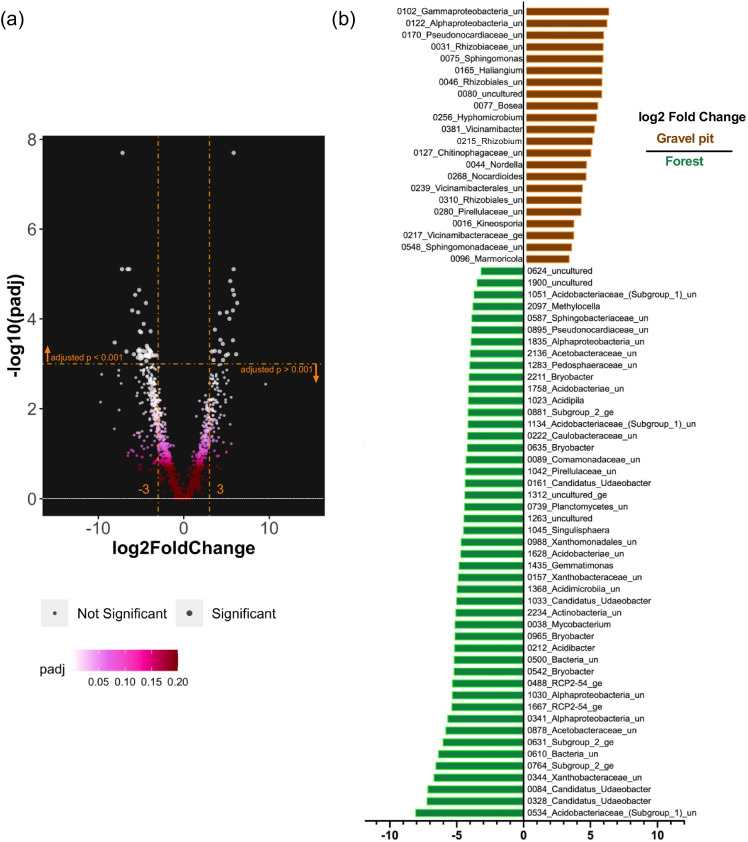
**a** Differentially abundant OTUs between the gravel pit and forest site represented as a volcano plot between absolute log2 fold change and adjusted p values determined through DESeq2 analyses. Bigger size dots represent significantly differentially abundant OTUs (adjusted p value <0.001). **b** Horizontal bar plot representing the 69 significantly differentially abundant OTUs between the gravel pit and forest site with adjusted p value <0.001 and absolute log2 fold change >3

Using DESeq2 analysis, we found 69 OTUs that were significantly differentially abundant between the gravel pit and the undisturbed forest site (adjusted p value <0.001) with an absolute log2 fold change >3 (Fig. 9a). Twenty-two of these OTUs were differently abundant at the gravel pit, of which approximately one-third belonged to the order *Rhizobiales*, including the genera *Rhizobium*, *Bosea*, *Hyphomicrobium* and *Nordella* (Fig. 9b). Whereas 47 OTUs were differently abundant at the forest site, dominated by the genera *Bryobacter* and Ca. *Udaeobacter* as well as the family *Acidobacteriaceae* (Subgroup 1) (Fig. 9b).

## Discussion

Little is known about the interaction of *Pinus contorta* with their bacterial microbiome in disturbed as well as undisturbed ecosystems, as most studies have employed culture-dependent methods (Anand et al. 2013; Padda et al. 2018). In addition, plant microbiome studies have largely concentrated on model and agricultural plants, with little focus on forest trees. In an attempt to comprehensively elucidate the rhizospheric and endophytic bacterial communities associated with lodgepole pine, we sampled young pine trees from a disturbed (Skulow gravel mining pit) and a nearby undisturbed (natural forest stand) site in central-interior BC. These sites differed significantly in soil characteristics, with the gravel pit having extremely low soil nutrient levels (particularly N and P), CEC and soil organic matter in comparison to the undisturbed forest site (Figs. 1 and 2, Table S1). Even for the forest site, the overall soil nutrient status was relatively low compared with other regions of the BC interior, likely due to the dry, cold climatic conditions and limited weathering (Driscoll et al. 1999; Sanborn et al. 2005, Kranabetter et al. 2006, Hope 2007). While significant differences in soil NH_4_^+^ levels were observed between gravel pit and forest site, the similar soil NO_3_^−^ levels observed at both sites suggests the abundance of nitrifying and denitrifying communities in the gravel pit soil, which remains to be examined.

Interestingly, the tissue N content of lodgepole pine trees at the gravel pit was unaffected by the dramatic differences in total N, available NH_4_^+^ and mineralizable N levels in gravel pit soils compared to the undisturbed forest soils. Other than these differences, there were no major environmental differences between the two sites in terms of N inputs and no N-fixing plant species were observed near the sampled trees. These findings are consistent with Kranabetter et al. (2006), Puri et al. (2018) and Chapman and Paul (2012), indicating that lodgepole pine tree growth is often unaffected by large differences in soil N levels. The combination of bare gravel substrate, lack of topsoil and low plant-available nutrients, make the Skulow gravel pit an extremely nutrient-poor environment, yet pioneering pines in the gravel pit have growth rates typical for the area. This suggests that pine trees could be relying on their microbiome to sustain their growth in an otherwise uninhabitable environment for conifers.

In this study, we determined the bacterial community structure of naturally regenerating pine trees post-disturbance in comparison to pines growing at the nearby undisturbed forest stand. By 16S rRNA sequencing, the composition and alpha and beta diversity indices of rhizosphere- and endosphere-associated microbiomes were characterized. All samples strongly clustered according to the site (gravel pit vs. undisturbed forest) and niche (rhizosphere, root, needle, stem) as evidenced from the Bray-Curtis beta diversity metrics (Fig. 7, Table 2). One possible explanation for these dissimilarities is that the site characteristics including the physical and chemical properties of soil influenced the differentiation of bacterial communities between both sites (Ullah et al. 2019; Firrincieli et al. 2020). Alternatively, host-dependent selection of certain communities as a result of adaptation mechanisms toward specific environmental constraints could be a major driver of variation (Laforest-Lapointe et al. 2016; Firrincieli et al. 2020). The site and niche differentiation in the tree bacterial microbiome have been previously observed in several poplar tree species including *Populus trichocarpa*, *P. deltoides* and *P. tremula* x *P. alba*, originating from sites with varying edaphic conditions (Gottel et al. 2011, Beckers et al. 2017, Firrincieli et al. 2020).

Our results indicate that the alpha diversity (Shannon index) of lodgepole pine bacterial communities inhabiting the aboveground tree tissues is lower in contrast to the belowground communities regardless of the origin site (Fig. 6). Rhizodeposition by the host plant including root exudation drives soil-plant-microbe interactions and enhances rhizosphere colonization, resulting in the structuring of a diverse microbiome in the rhizosphere (Bais et al. 2006; Beckers et al. 2017). A decrease in alpha diversity in the aboveground plant parts is in line with previous studies (Zarraonaindia et al. 2015; Deyett and Rolshausen 2020), suggesting that the rhizosphere-root interface acts as a bottleneck to the bacterial richness, and that the ability to colonize aerial plant tissues is limited to specific bacteria. Systemic colonization of lodgepole pine by certain bacteria as indicated by the proportion of OTUs shared within all plant niches (gravel pit: 16.5%, undisturbed forest: 27.2%, Fig. 5) also highlights that many bacterial endophytes likely originate in the rhizosphere, penetrate plant root cells, and reach the xylem vessels to colonize internal tissues of the host plant (Compant et al. 2005, 2010).

The proportion of OTUs uniquely identified in the aboveground niches (5–6%) (Fig. 5) indicates that, while most endophytic bacteria likely originate from the rhizosphere soil (Compant et al. 2010), some may gain entry to the tree via vertical transmission from seeds (Frank et al. 2017) or horizontal transmission routes including wounds (Munkvold and Marois 1995) or natural openings such as stomata (Fahlgren et al. 2010; Compant et al. 2011). In addition, each plant niche offers distinct microenvironments (Bulgarelli et al. 2013; Beckers et al. 2017) which may explain why certain proportions of OTUs are confined to specific niches only (Fig. 5).

At the phylum level, *Proteobacteria* (mainly *Alphaproteobacteria*) dominated the bacterial assemblages at both the gravel pit and undisturbed forest site (Figs. 3a and S2) possibly due to their ability to respond to labile C sources, rapid growth, and adaptation to diverse plant niches (Lagos et al. 2015). After *Proteobacteria*, to a lesser extent, the *Actinobacteria* and *Acidobacteria* were predominant at the gravel pit and forest site, respectively (Figs. 3a and S2). The dominance of *Acidobacteria* at the forest site could be due to their high metabolic versatility that allows them to decompose complex C substrates present in the forest ecosystem (Rasche et al. 2011; Naether et al. 2012). In addition, *Acidobacteria* have also been linked to dissimilatory nitrate reduction to ammonium, mobilization of ammonium in soils and N-fixation processes, which may explain the higher NH_4_^+^ content in undisturbed forest soils (Kielak et al. 2016; Eichorst et al. 2018; Kalam et al. 2020). Interestingly, the RA of *Proteobacteria* increased from the rhizosphere soil to endosphere niches and the RA of *Actinobacteria* and *Acidobacteria* decreased from the rhizosphere to endosphere (Fig. 3a), a trend which has also been observed in microbiomes of grapevine, poplar and rice (Gottel et al. 2011, Beckers et al. 2017, Deyett and Rolshausen 2020). In terms of unique phyla between both sites, *Chloroflexi* and *Firmicutes* were primarily detected in the rhizosphere and *Cyanobacteria* in the needle tissues of pine trees at the gravel pit (Fig. 3a). Species within *Chloroflexi* and *Firmicutes* phyla have been associated with stress tolerance such as low nutrient concentrations and limited labile carbon (Uroz et al. 2016; Fierer 2017), much like the conditions at the gravel pit. In addition, genera of phyla *Firmicutes* have been closely linked to N cycling processes including N-fixation, nitrification, and denitrification (Feng et al. 2015; Srivastava et al. 2016; Puri et al. 2017b). Furthermore, *Chloroflexi* was also associated with nitrification activity in a previous study (Sorokin et al. 2012). *Cyanobacteria* are known for their photosynthetic and N-fixing capacity and have been previously observed in aboveground tissues of various plant species including wheat (Gantar et al. 1995), potato (Ringelberg et al. 2012), Jingbai pear (Ren et al. 2019a), and Norway spruce (Ren et al. 2019b) as well as several vascular plant species in a Costa Rican rainforest (Fürnkranz et al. 2008).

Though the association of plant-beneficial endophytic bacteria with lodgepole pine has been extensively studied using culture-dependent methods (Anand et al. 2013; Puri et al. 2017a, 2020a, b), no study so far has examined the complete bacterial microbiome of pine using high throughput sequencing. In the past, studies have only focused on analyzing the microbiome of a particular niche of lodgepole pine. For example, two decades ago, Chow et al. (2002) evaluated the rhizosphere bacterial communities of lodgepole pine at three Long-term Soil Productivity (LTSP) sites in central BC (one site within 1 km distance of our study sites) with varying levels of disturbance including surface organic matter removal and soil compaction. Post-disturbance, *Alphaproteobacteria* and *Actinobacteria* dominated the pine rhizosphere communities at the LTSP sites (Chow et al. 2002). These results correspond with our observations of pine trees at the gravel pit, suggesting a possible pattern in the assembly of pine rhizosphere bacterial community after disturbance events. In addition to this, Carrell et al. (2016) evaluated the needle bacterial communities of lodgepole pine in nutrient-limited subalpine ecosystems of Colorado and California, USA and identified *Alphaproteobacteria* (particularly, *Acetobacteraceae*) as the most dominant taxa followed by *Bacteroidetes*.

The composition and proportion of root and rhizosphere bacterial microbiota varied substantially between the gravel pit and undisturbed forest site, where Proteobacterial families (mainly *Rhizobiaceae, Comamonadaceae, Xanthobacteraceae*) evenly enriched the belowground communities at the gravel pit while *Xanthobacteraceae* alone was more predominant at the forest site (Figs. 3b and 4). Members of the family *Rhizobiaceae* and *Comamonadaceae* have been reported to typically associate with pioneering plants in oligotrophic environments of disturbed areas, such as the post-mining initial-development reclamation sites in Germany (Vuko et al. 2020), the oil sands reclamation sites in northern Alberta, Canada (Mitter et al. 2017) and the LTSP sites in central BC (Chow et al. 2002). These results suggest that in nutrient-poor post-disturbance settings, plants likely associate with these well-known beneficial microorganisms for their survival and growth. Furthermore, in the belowground root and rhizosphere niches at the gravel pit, two OTUs from the *Comamonadaceae* group and three OTUs from the *Rhizobiaceae* group (including notable N-fixers *Rhizobium* and *Mesorhizobium*) were among the most abundant OTUs with RA of approx. 15% (Fig. 8). Members of *Xanthobacteraceae* have been found to associate with the rhizosphere and roots of rice (Chang et al. 2021) and tea plantations (Chen et al. 2021). In addition, bacteria within the families *Rhizobiaceae, Comamonadaceae,* and *Xanthobacteraceae* have been closely linked to N cycling processes including N-fixation, nitrification, and denitrification in the past studies (Chen and Ni 2011; Gomez-Alvarez et al. 2014; Jang et al. 2020), which might help explain the soil N dynamics of the gravel pit.

The aboveground (stem and needle) community varied substantially from the belowground (root and rhizosphere) community, implying some degree of microbial selection or adaptation to plant niches. The composition of the stem and needle microbiome of lodgepole pine was very similar across the gravel pit and undisturbed forest site (Figs. 3b and 4), in addition to being comparable to the endo-microbiome of other *Pinaceae* species in nutrient-limited environments (Carrell and Frank 2014; Carrell et al. 2016; Moyes et al. 2016; Carper et al. 2018). Two Proteobacterial families, *Acetobacteraceae* and *Beijerinckiaceae*, dominated needle and stem niches at both sites, with five OTUs of the *Acetobacteraceae* and six OTUs of the *Beijerinckiaceae* among the 50 most abundant OTUs in our dataset (Fig. 8). The large overlap in key community members of aboveground endophytic bacterial assemblages across both sites demonstrates that: (i) efficient endophytic colonization of specific plant niches is potentially reserved for a minority of bacterial taxa, and/or (ii) dominant stem and needle bacterial communities of lodgepole pine are likely seed-borne instead of soil- or air-borne because it is likely that the seed source for gravel pit trees is the nearby undisturbed forest site (Carrell et al. 2016; Beckers et al. 2017).

The consistent dominance and coexistence of distinct *Acetobacteraceae* OTUs have been previously reported in foliage endophytic communities of Engelmann spruce (*Picea engelmannii*) and limber pine (Carrell and Frank 2014; Carper et al. 2018), suggesting a possible selection and mutualism between members of the *Pinaceae* family and *Acetobacteraceae* endophytes. One phylotype (OTU_4) with 48% RA in lodgepole pine stem tissues at the gravel pit shared 100% similarity with one of the most abundant OTUs of limber pine (Carper et al. 2018) and lodgepole pine in the western US (Carrell et al. 2016). Taken together, these results suggest that acetic acid bacteria are likely core members of the endophytic microbiome across diverse *Pinus* host species and locations. Furthermore, Moyes et al. (2016) proposed that foliar endophytic bacteria, particularly members of the *Acetobacteraceae* may be involved in fixing N endophytically based on the ^13^N radioisotope enrichment and acetylene reduction assays performed on limber pine twig samples. Endophytic and rhizospheric members of this acetic acid bacterial family, including *Gluconacetobacter*, *Acetobacter*, *Asaia* and *Swaminathania*, are known to fix N in association with sugarcane (*Saccharum officinarum*), rice (*Oryza sativa*), sweet potato (*Ipomoea batatas*), Kombucha tea (*Medusomyces gisevii*) and coffee (*Coffea arabica*) (Boddey et al. 1991, 2001; Pedraza 2008; Saravanan et al. 2008; Komagata et al. 2014; Reis and Teixeira 2015). However, it is important to note that the link between these acetic acid bacteria and N fixation needs to be further explored using more robust methods such as shotgun metagenome sequencing before firm conclusions can be drawn. Besides *Acetobacteraceae*, *Beijerinckiaceae* also dominated aboveground pine niches at both sites, similar to what was observed in limber pine and Engelmann spruce foliage in nutrient-poor subalpine environments (Carrell and Frank 2014; Marín and Arahal 2014). Previous studies have reported that members of the *Beijerinckiaceae* family associate with conifers such as *Lepidothamnus fonkii* inhabiting N-deficient ombrotrophic peatlands (Borken et al. 2016) and Corsica pine (*Pinus nigra*) originating from the sandy soils of Culbin forest in Scotland (Izumi et al. 2006).

Previously, we characterized the culturable endophytic bacteria associated with lodgepole pine trees at the Skulow gravel pit which exhibited significant potential to stimulate host-tree growth through several mechanisms including N-fixation, phosphate solubilization, phytohormone modulation and siderophore production (Padda et al. 2018, 2019, 2021). In a year-long greenhouse study, these culturable endophytes of genera *Pseudomonas*, *Rhizobium* and *Flavobacterium* fixed significant amounts of N *in planta* (up to 53%), estimated using a ^15^N isotope dilution assay (Padda et al. 2019). However, the culturable bacteria represent a very small proportion of the tree bacterial microbiome and likely don’t elucidate all the functions of the entire bacterial community. Nevertheless, the dominant bacterial taxa observed in all pine niches in the current study, particularly phylotypes of the *Rhizobiaceae*, *Acetobacteraceae*, *Xanthobacteraceae* and *Beijerinckiaceae*, also imply that lodgepole pine trees could be associating with beneficial bacteria at the disturbed gravel pit. Furthermore, the prevalence of *Rhizobiales* bacteria (well-known beneficial partners in plant-microbe interactions) as the differentially abundant taxa at the gravel pit in comparison to the undisturbed forest site (Fig. 9), demonstrates the potential dependence of lodgepole pine on its bacterial microbiome for survival and growth under nutrient-poor conditions. Nonetheless, it is important to study the N cycling genes in soil and plant environments to help explain how these conifers can grow in severely N-limited gravel substrate and from where pine trees are accumulating the unknown N in their tissues. Furthermore, the lack of available NH_4_^+^ in gravel pit soils but identical available NO_3_^−^ levels in gravel pit soils and undisturbed forest soils, also raises the likeliness of nitrification occurring at the gravel pit which should be investigated further.

Notwithstanding the potentially important role prokaryotes play in pine growth at nutrient poor sites, the influence of the fungal microbiome, especially root-associated mycorrhizal fungi, in supporting the growth of gravel pit pine trees cannot be ignored. Isolates of the genera *Rhizopogon*, *Suillus*, *Laccaria*, *Hebeloma*, and *Scleroderma* have been reported to enhance the growth of *Pinus* trees through nutrient scavenging in soils and plant growth hormone modulation (MacFall and Slack 1991; Scagel and Linderman 1998; Ortega et al. 2004). Therefore, we are currently evaluating the composition and possible roles of the fungal microbiome of lodgepole pine trees at the Skulow gravel pit to explore the importance of fungal communities in sustaining pine growth at disturbed environmental sites.

In summary, this study provides the first comprehensive analysis of the bacterial microbiome of lodgepole pine trees under varying soil conditions. The profile of rhizosphere and endosphere microbiota highlight diverse bacterial communities with potential plant growth-promoting capabilities. The current study helps improve our understanding of the native tree microbiome in boreal ecosystems and the natural revegetation strategies of long-lived conifers on disturbed sites. It is recommended that future studies should focus on using shotgun metagenomics and metaproteomics to move from the descriptive phase of studying the tree microbiome and assign functions to the members of the community.

## Supplementary Information

ESM 1 (HTML 273 kb)

ESM 2 (PDF 599 kb)
