# Supplementary material for: Evaluating the rhizospheric and endophytic bacterial microbiome of pioneering pines in an aggregate mining ecosystem post-disturbance
Source: Plant Soil. 2022 Mar 9;474(1-2):213–32. doi: 10.1007/s11104-022-05327-2 (PMC9184430; doi:10.1007/s11104-022-05327-2)
Supplement: Supplementary file 1 — (HTML 273 kb) [file 11104_2022_5327_MOESM1_ESM.html]

Javascript must be enabled to view this page.

magnitude
magnitudeUnassigned

Forest
Gravelpit

407195316754

407195316754

15668

15668

15668

15668

15668

577

577

577

577

577

44805

44805

44805

44805

44805

2916

2916

2916

2916

2916

128765

128765

128765

128765

128765

6034636

81375

102

102

102

11230

11230

11230

743

743

743

4502477

4502477

4502477

4502477

58598

58598

58598

58598

87186

87186

87186

87186

134

134

134

134

134

3408841861

2881555

2881555

2881555

2881555

3290337933

31661470

2051187

2051187

228366

228366

1103

1103

22432

22432

864382

70247

794135

11320

6511

6511

489

489

4722048

4722048

4231794

49254

4753564

42

42

4692609

13788

52350

165744

1151427

6913

6913

11306221

11306221

3462991

7843230

31465779

31465779

42

543773

8871411

17163553

33737446

7305

7186

119

33294540

33174478

1262

371543

101507

2736

1058

1058

4193618

4193618

4193618

2520865

2520865

2520865

180896902

180896902

180896902

727

727

727

727

8902346

232105

232105

232105

1729

1729

1729

1661938

23250

23250

1411454

97535

19849

2570

2234

2234

475274

475274

475274

9671140

9671140

9671140

9671140

1651

31334

4891105

18722262

110336

110336

110336

75263

3573

17521804

823548

13126

13126

582351

582351

22871

20032

2839

4721042

4721042

4721042

457214

457214

457214

10122

10122

10122

10122

2321102

925

925

925

925

1401097

9822

9822

9822

131275

131275

131275

6919723012

6065314203

3088453

3088453

3088453

67256

67256

67256

6444254

6444254

6444254

4992813279

1949172

1949172

3332205

3332205

4464712902

47021124

64802565

128263401

76286

198775726

521161

521161

521161

12421022

147539

147539

120309

17195

1035

37

37

37

1058483

1058483

1058483

73027787

72837233

76440

76440

68444484

68444484

3632309

40

681

3631588

19554

19554

19554

243694205904

322279

322279

322279

322279

2921726869

4293451

4293451

4293451

557

557

557

1291565

1291565

1291565

1567218251

493713504

477713204

160300

4981315

123

405753

398

9341

7284

7284

300298

300298

936749

936749

8076797

7615605

456135

557

8531504

260767

593737

46561808

46561808

46561808

7069

7069

7069

6286556

6286556

6286556

15041109

704281

600250

10431

523822

12431

45145

353105

1541

2776

2776

4663

4663

4663

214155178756

388141

388141

388141

1917804

1917804

1465

1903739

6461059505

6461059505

10526

3316653494

96272999

16211561

20186925

6312

6312

6312

290416700

290416700

223612903

2146

1243264

542387

12439191032

282911

282911

223919317

341319

13345802

6314756

2407440

4820031032

984

193

8694967

3880819560

10860

5828068

71480

10533595

473969

5802626

6393715937

1574

149217326

420286245

6635569

1961793

91392

91392

766914553

766914553

8781688

140239

7381449

423607

4801

91296

291510

354

354

78

276

121755797

121755797

121755797

97266

97266

97266

67354345

67354345

1213500

17221349

2642856

1006488

1521152

1924912526

1924912526

1924912526

1924912526

1924912526

687614221

687614221

33524765

33524765

8637

655784

5733

160382

23943529

250263

3627

3627

195189

152112

2638

1739

1947

1947

100539

100539

100539

3537462

148226

148226

1957173

1957173

1063

1063

28211192

28211192

359759

55227

2407206

84303939

63973452

3201907

3201907

3091668

11239

3492322

3492322

973201

151682

100339

295142

29588

29588

54

54

478

478

478

1870480

1870480

1870480

416523

416523

416523

1874400

1874400

1874400

1874400

15987

15987

15987

15987

194113280

1143

1143

1143

1143

194003149

680172

680172

680172

170462380

1422409

1422409

156241971

156241971

1607243

1607243

19454

1413189

67354

67354

67354

88

88

88

88

14557

518

518

518

518

1439

1439

1439

1439

1045749

936667

778482

3461

3461

718372

718372

2649

2649

6719

6719

6719

91166

89120

89120

246

246

10982

10982

10982

10982
