# Supplementary material for: Evaluating the rhizospheric and endophytic bacterial microbiome of pioneering pines in an aggregate mining ecosystem post-disturbance
Source: Plant Soil. 2022 Mar 9;474(1-2):213–32. doi: 10.1007/s11104-022-05327-2 (PMC9184430; doi:10.1007/s11104-022-05327-2)

Evaluating the rhizospheric and endophytic bacterial microbiome of pioneering pines in an aggregate mining ecosystem post-disturbance

*Kiran Preet Padda[a, *], Akshit Puri[a, b, c, *], Nguyen Khoi Nguyen[d], Timothy J Philpott[e] and Chris P Chanway[a]*

[a] Department of Forest and Conservation Sciences, Faculty of Forestry, The University of British Columbia, Vancouver, BC, Canada.

[b] School of Agriculture and Food Science, University College Dublin, Belfield, Dublin 4, Ireland

[c] UCD Earth Institute, University College Dublin, Belfield, Dublin 4, Ireland

[d] Microbiome Insights Inc., Vancouver, Canada.

[e] British Columbia Ministry of Forests, Lands and Natural Resource Operations, Williams Lake, BC, Canada

**Corresponding authors.*

SUPPLEMENTARY DATA

Table S1 Soil properties and concentrations of macro- and micro-nutrients determined for the mineral soil samples (0–20 cm depth; n = 20) collected from the gravel pit and forest site.

	**Gravel pit**	**Forest**
Available Phosphorus (mg/kg)	< DL	39.20 ± 10.84
Organic matter (%)	1.160 ± 0.051***	10.06 ± 1.242
Organic carbon (%)	0.673 ± 0.030***	5.835 ± 0.720
pH (H_2O)	8.340 ± 0.040***	5.520 ± 0.213
pH ($CaCl_2$)	7.700 ± 0.001***	4.940 ± 0.229
Cation exchange capacity (cmol+/kg)	10.50 ± 0.447*	19.20 ± 2.800
Sand (%)	80.60 ± 2.786***	58.60 ± 2.909
Silt (%)	12.16 ± 1.267***	32.80 ± 2.728
Clay (%)	7.240 ± 1.694	8.940 ± 0.273
Soil texture	Loamy Sand	Sandy Loam
Macro- and Micro-nutrients (mg/kg)		
Aluminum	318.0 ± 15.93***	638.0 ± 40.67
Boron	0.840 ± 0.214	1.240 ± 0.075
Calcium	3180 ± 188.1**	1820 ± 239.6
Copper	2.900 ± 0.277***	0.330 ± 0.220
Iron	306.0 ± 20.40***	498.0 ± 17.72
Potassium	36.80 ± 4.576***	156.0 ± 9.274
Magnesium	360.0 ± 35.77*	646.0 ± 92.55
Manganese	83.00 ± 5.683	103.0 ± 16.53
Sodium	< DL	< DL
Phosphorus	< DL	83.00 ± 20.25
Sulphur	4.600 ± 2.821*	14.20 ± 1.020
Zinc	< DL	6.100 ± 1.738

* $P < 0.05$; ** $P < 0.01$; *** $P < 0.001$ (significantly different from forest site)
< DL = Less than estimated detection limit (Available Phosphorus: 1 mg/kg; Sodium: 10 mg/kg; Phosphorus: 20 mg/kg; Zinc: 2 mg/kg)

Table S2 Concentration of various nutrients in the lodgepole pine tree samples collected from the gravel pit and forest site (n = 5).

Macro- and Micro-nutrients (mg/kg)	Gravel pit	Forest
Aluminum	829.0 ± 264.0	578.0 ± 165.4
Boron	15.20 ± 3.794	13.00 ± 1.660
Calcium	3490 ± 510.2	2810 ± 297.2
Copper	8.970 ± 2.093	5.870 ± 1.124
Iron	1121 ± 387.3	689.9 ± 269.9
Potassium	3750 ± 314.2	3300 ± 177.6
Magnesium	1600 ± 219.1	1360 ± 103.5
Manganese	108.2 ± 9.620***	268.0 ± 33.09
Molybdenum	< DL	0.276 ± 0.200
Sodium	69.60 ± 40.99	7.800 ± 5.200
Phosphorus	987.0 ± 60.44	836.0 ± 51.70
Sulphur	287.0 ± 118.6	382.0 ± 105.6
Zinc	44.70 ± 4.384	50.10 ± 4.401

*** $P < 0.001$ (significantly different from forest site)
< DL = Less than estimated detection limit (Molybdenum: 0.5 mg/kg)

Bacterial community–Soil properties Interaction Methodology:

To study the association between bacterial microbiome and soil properties we fitted different soil elements and the ordination of the microbiome data on linear regression models using the *enfit* function from the *vegan* package (Oksanen et al. 2020). Soil properties were treated separately as a dependent variable that could be explained by ordination scores (PC1 & PC2) from the microbiome data. R-squared (goodness of fit) and p-value of permutation test (999 times) were obtained for each model and significance was determined when the p-value was less than 0.05. Each soil variable was presented as an arrow, where the direction of the arrow shows an increasing gradient. The length of the arrow is proportional to the correlation between the soil variable and the ordination of the microbiome. In order to obtain the ordination of the bacterial microbiome, raw count data at different taxonomic levels was transformed using phylogenetic isometric log-ratio implemented by *philr* package (Silverman et al., 2017) to account for compositionality. The transformed data was then used to calculate principal component analysis and visualized by the *fviz_pca_var* function in the factoextra package (Kassambara and Mundt, 2020). Each arrow is a balanced node determined by a voting scheme for the two clades (A/B) that descend from a given balance. A is the numerator of the log-ratio and B refers to the denominator identified by *name.balance* function from *philr* package (Silverman et al., 2017). The contribution in percentage of each node to the ordination is estimated as the quality of representation, which is the squared coordinate (x^2, y^2) over the total quality of representation of all nodes (Kassambara and Mundt, 2020).

References

Kassambara, A., Mund, F., 2020. factoextra: Extract and Visualize the Results of Multivariate Data Analyses. R package version 1.0.7. Available from: https://CRAN.R-project.org/package=factoextra. accessed 26.09.2021.

Oksanen, J., Blanchet, F.G, Friendly, M., Kindt, R., Legendre, P., McGlinn, D., Minchin, P.R., O'Hara, R.B., Simpson, G.L., Solymos, P., Henry, M., Stevens, H., Szoecs, E.,and Wagner, H., 2020. Vegan: Community Ecology Package. R package version 2.5-7. Available from: https://CRAN.R-project.org/package=vegan. accessed 26.09.2021.

Silverman, J.D., Washburne, A.D., Mukherjee, S., David, L.A., 2017. A phylogenetic transform enhances analysis of compositional microbiota data. eLife 6, e21887.

Fig. S1. Principal component (PC) analysis showing correlation between major soil parameters and the bacterial community data (OTUs) evaluated using phylogenetic isometric log-ratio (see methodology below) for the (a) gravel pit and (b) forest site. Soil parameters are indicated by blue and green arrows in PC charts for gravel pit and forest site, respectively. R-squared (r^2) and P values pertaining to the correlation of each soil parameter with bacterial community data are summarized in tables. TN: total nitrogen; TC: total carbon; pH_H_2O: pH in water; pH_$CaCl_2$: pH in calcium chloride; AA: available ammonium; AN: available nitrate; MN: mineralizable nitrogen; AP: available phosphorus; CEC: cation exchange capacity; SOC: soil organic carbon. * $P < 0.05$ (significant association between soil parameter and bacterial community).

(a) Gravel pit

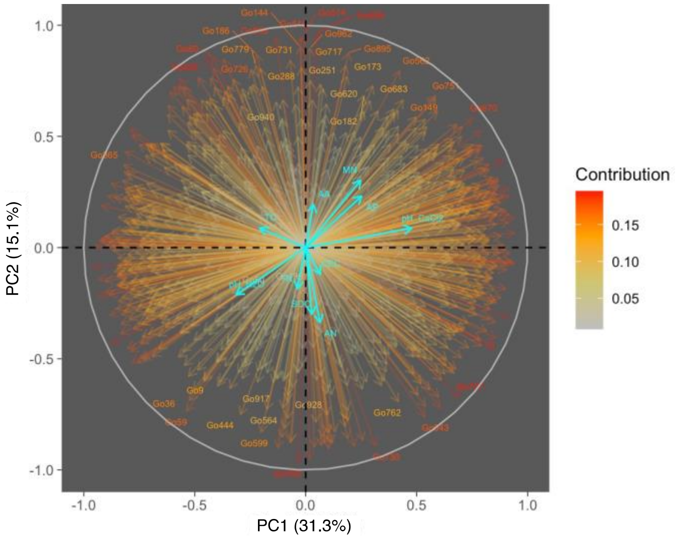

	PC1	PC2	r^2	Pr (>r)
**TN**	-0.198	-0.980	0.036	0.770
**TC**	-0.921	0.388	0.051	0.669
**pH_H_2O**	-0.829	-0.559	0.143	0.316
**pH_$CaCl_2$**	0.984	0.179	0.236	0.111
**AA**	0.178	0.984	0.039	0.780
**AN**	0.189	-0.982	0.118	0.406
**MN**	0.634	0.773	0.153	0.293
**AP**	0.736	0.677	0.117	0.382
**CEC**	0.480	-0.877	0.019	0.851
**SOC**	0.094	-0.996	0.091	0.468

(b) Forest

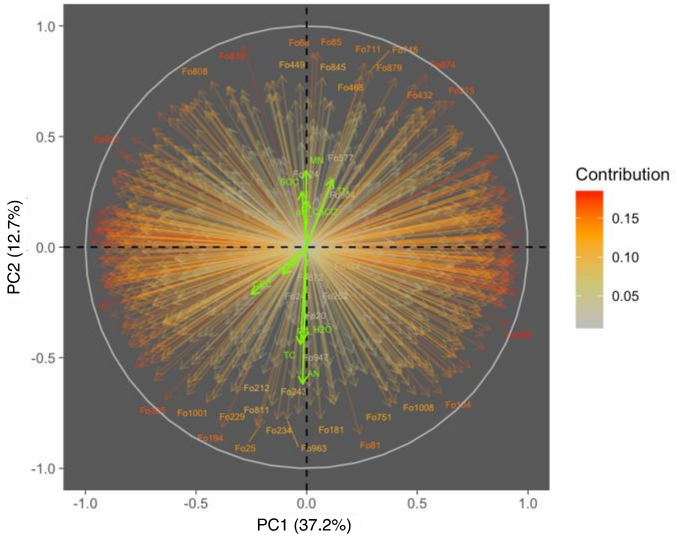

	PC1	PC2	r^2	Pr (>r)
**TN**	0.356	0.935	0.107	0.466
**TC**	-0.065	-0.998	0.193	0.210
**pH_H_2O**	-0.031	-0.999	0.174	0.246
**pH_$CaCl_2$**	-0.011	0.999	0.045	0.697
**AA**	-0.670	-0.743	0.027	0.813
**AN**	-0.029	-0.999	0.383	**0.035***
**MN**	-0.006	0.999	0.119	0.385
**AP**	0.602	-0.799	0.007	0.955
**CEC**	-0.760	-0.650	0.107	0.459
**SOC**	-0.087	0.996	0.063	0.626

Fig. S2. Krona chart representing taxonomic composition of the whole bacterial community associated with lodgepole pine at the disturbed gravel pit and undisturbed forest site. See supplementary file (Fig. S2 – Krona chart.html) for the interactive version of the Krona charts.

Fig. S3. Relative abundance of bacterial (a) class and (b) orders within individual lodgepole pine niches (needle, rhizosphere, root and stem) at the gravel pit and forest site. Low-abundance classes and orders (< 4% relative abundance) represent the unfilled portion of the bar plots.

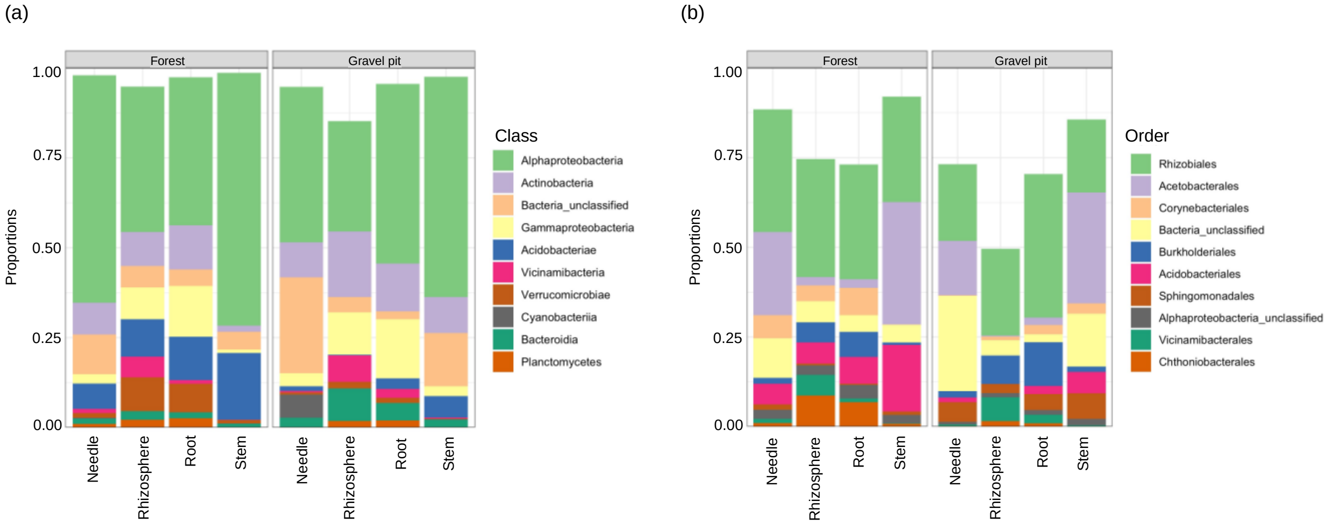

Supplement: Supplementary file 2 — (PDF 599 kb) [file 11104_2022_5327_MOESM2_ESM.pdf]
